# Area deprivation levels of members of a fully digital diabetes prevention program compared to the US population

**DOI:** 10.3389/fendo.2025.1597945

**Published:** 2025-07-16

**Authors:** Sarah K. Pickus, Kimberly G. Lockwood, Sarah A. Graham

**Affiliations:** Clinical Research & Data Science, Lark Health, Mountain View, CA, United States

**Keywords:** area deprivation index, type 2 diabetes, prediabetes, DPP uptake, mHealth

## Abstract

**Background:**

Deprived geographic areas have high rates of poverty, unemployment, low education, and limited access to health care, which can lead to poor health outcomes. Fully digital deliveries of the National Diabetes Prevention Program (DPP) can offer accessible preventive healthcare to individuals living in deprived areas, but this does not necessarily mean that these individuals will participate in digital health solutions. There is a pervasive belief that digital solutions are only used by socially advantaged groups.

**Objective:**

Determine whether a fully digital DPP has uptake in individuals residing in areas with high deprivation levels to substantiate that digital solutions can help overcome barriers to diabetes care.

**Methods:**

An observational study comparing area deprivation levels of N=41,375 digital DPP members with commercial insurance and N=844 with Medicaid to that of US adults with either commercial insurance or Medicaid coverage. Data sources included demographic and geographic data from members enrolled during or after 2016 in the digital DPP, the 2020 Area Deprivation Index (ADI) v3.2 dataset, and Table B27010 from the 2016–2020 American Community Survey.

**Results:**

Digital DPP members with commercial insurance lived in areas with higher deprivation than the commercially insured US population, *D*=0.13, *p*<.001. ADI quintile 1 (least deprived) represented 14.7% of digital DPP members vs. 22.5% of the US population; quintile 5 (most deprived) represented 19.2% of digital DPP members vs. 13.2% of the US population. Digital DPP members with Medicaid coverage lived in areas with higher deprivation than the comparable Medicaid-covered US population, *D*=0.35, *p*<.001. ADI quintile 1 represented 0.4% of digital DPP members vs. 16.7% of the Medicaid population, and quintile 5 represented 50.0% of digital DPP members vs. 26.6% of the Medicaid population.

**Conclusions:**

This study demonstrates that a digital DPP had uptake in individuals with prediabetes who lived in areas with higher deprivation than the comparable US population. This finding suggests that a digital DPP indeed reached individuals in high deprivation areas in need of accessible preventive healthcare, contradicting the stereotype that digital solutions only reach socially advantaged individuals with prediabetes. Providing much-needed, on-demand care may help to mitigate the associated negative health impacts of living in a deprived area.

## 1 Introduction

The Centers for Disease Control and Prevention (CDC) defines health equity as “the state in which everyone has a fair and just opportunity to attain their highest level of health” (1). Socially determined circumstances should not impede one’s access to or quality of health care. However, despite ongoing strategies to mitigate their influence, social determinants of health (SDOH) are strongly associated with health risks and outcomes (2, 3). Where you live, work, and spend your time impacts your health.

The Area Deprivation Index (ADI) is a measure of social disadvantage at the census tract level, with higher values indicating higher poverty and disadvantage (4). Deprived areas have high rates of poverty, unemployment, low education, and limited access to health care, which can lead to poor health outcomes (4). For example, areas with greater deprivation tend to have a higher prevalence of risk factors for diabetes, such as obesity, high blood pressure, poor diet and nutrition, and lack of physical activity, which increase the likelihood of chronic conditions such as diabetes (5, 6). Indeed, there is a higher prevalence of prediabetes and diabetes in people living in deprived areas (7–9).

In addition to a higher prevalence of diabetes in deprived areas, patients living in more deprived and rural areas are less likely to attain high-quality diabetes care compared with those living in less deprived and urban areas (10). Kurani and colleagues (11) observed that patients living in the quintile of counties with the highest ADI had a 41% higher risk of emergency department visits and hospitalizations due to severe hypoglycemia and a 12% higher risk due to diabetic ketoacidosis or hyperglycemic crisis than those living in the least deprived quintile of counties. Also notable is that individuals at high clinical risk living in neighborhoods with the greatest disadvantage incur significantly greater healthcare costs than those living in the least disadvantaged areas (12). One reason for these collective findings may be a lack of access to adequate diabetes prevention and management resources. For example, Jayapaul-Philip and colleagues (13) found that lifestyle change programs geared toward diabetes prevention were clustered in counties with low diabetes incidence and high socioeconomic status rather than in areas with high diabetes incidence or low socioeconomic status. There is a clear need to provide tools for improved diabetes prevention and management to people residing in areas with high deprivation.

Digital health programs can offer disease prevention and management support to individuals experiencing geographic and social barriers to care (14–16). However, the fact that remote delivery of digital programs increases their accessibility does not necessarily mean that individuals living in deprived areas will participate in digital care offerings. Although research has demonstrated the benefits associated with using digital technologies, such as enabling patients to better manage their health, there is still concern that digital technologies are only accessible to, or usable by, individuals from higher socioeconomic backgrounds that have less need for these tools (17). Digital health programs need to demonstrate uptake with individuals living in deprived areas to substantiate that digital solutions can help overcome barriers to diabetes care.

Previous research demonstrates that fully digital deliveries of the National Diabetes Prevention Program (DPP) help extend the program’s reach (18) and can provide much-needed preventive health care to individuals living in areas with insufficient numbers of healthcare practitioners (19). The purpose of this study was to extend upon this body of work by exploring whether a fully digital DPP has uptake in individuals residing in areas with high deprivation and can thus help to address barriers to participation in onsite programs. This study compared the ADI of members participating in a fully digital delivery of the National DPP to that of US adults with commercial insurance or Medicaid coverage, respectively. The primary hypothesis was that individuals enrolled in the digital DPP would have a higher ADI relative to US adults with commercial insurance but that this relationship would not necessarily be true for those covered by Medicaid insurance, due to the known SDOH affecting the Medicaid population. The secondary hypothesis was that digital DPP members living in areas with a high ADI would have higher body weights, consistent with the elevated risk factors for diabetes observed in published literature.

## 2 Methods

### 2.1 Lark digital DPP

The Lark DPP is a digital lifestyle change program that has full recognition from the Centers for Disease Control and Prevention (CDC) and follows the PreventT2 curriculum (20). A supplement describing the program in detail is published elsewhere (21). Briefly, members of the program do not need to have an official diagnosis of prediabetes but they must be at high risk for type 2 diabetes based on CDC-defined inclusion criteria and risk assessment (22). In addition to the weekly educational lessons of the PreventT2 curriculum (26 in total), members of the program engage with personalized coaching powered by artificial intelligence (AI); log and track meals, physical activity, and body weight; and complete screeners related to other areas of preventive health care (e.g., depression). Members access the program via an application downloaded on their iOS or Android smartphone, interact with the AI-powered platform via a text message-like interface, and receive a cellular-connected body weight scale that automatically uploads their weights to the Lark application. The program is 12 months long and has a primary goal of members achieving 5% weight loss. Papers on the performance and outcomes associated with the Lark digital DPP can be found elsewhere (21, 23–25).

### 2.2 Ethical approval

This study received exemption status from Advarra (Protocol #Pro00047181) Institutional Review Board for retrospective analyses of previously collected and de-identified data. All Lark members agreed to a privacy policy at registration, which included permission to use their de-identified data for research.

### 2.3 Area deprivation index dataset

The ADI is a composite measure of 17 census variables that describe socioeconomic disadvantage based on income, education, household characteristics, and housing (4). ADI values range from 1 to 100, with 100 representing communities with the most deprivation. ADI values used in this paper came from the 2020 ADI v3.2 dataset (4, 26), which uses estimates from the 5-year 2016–2020 American Community Survey (ACS) and 2020 decennial Census. The ADI dataset contains a national percentile of block group ADI value, a state-specific decile of block group ADI value, the block group Census ID, and a key linkage field to the block group shapefile served by the National Historical Geographic Information System (NHGIS). This study used the national percentile block group ADI values.

### 2.4 Participants

#### 2.4.1 Participants with commercial insurance

The first analysis of individuals with commercial insurance compared a large sample of N=45,076 members who enrolled during or after September 2016 in the digital DPP to the broader US population. Members included in the digital DPP sample had to have full address information available to geocode their data and calculate their respective ADI values. Digital DPP members represented all 10 Health and Human Resources (HHS) regions, enabling comparison to the entire available US population. After filtering out members who belonged to 226 census blocks with ADI suppression codes (0.7% of the available block groups in this sample), this resulted in a final population of N=41,375 digital DPP members with commercial insurance. ADI suppression codes mean that the ADI values for certain areas are not available, and reasons include low population and/or housing, a high population living in group quarters, or meeting both criteria.

The commercially insured comparator group contained N=191,341,161 US adults aged ≥19 years with health insurance that did not include Medicaid. Table B27010 from the 2016–2020 ACS (27) provided population estimates at the block group level segmented by age and health insurance coverage. Using these data, we estimated a total of 24,042,068 uninsured adults, or 9.9% of the US adult population, and removed them from the commercial insurance analyses. We confirmed the accuracy of this estimate by calculating the percentage of adults and children without health insurance and finding this estimate to be 8.7% of the US population, very close to the 8.6% published by the Census Bureau for 2020 (28). We further removed individuals with Medicaid/means-tested public coverage, or two or more types of health insurance coverage, including Medicare and Medicaid/means-tested public coverage (described below in the Medicaid section). After filtering out members who belonged to 3,342 census blocks with ADI suppression codes (1.4% of the available block groups in this sample), this resulted in a final population of N=188,609,136 US adults with commercial insurance, or 77.3% of the US adult population.

#### 2.4.2 Participants with Medicaid coverage

The second analysis of individuals with Medicaid coverage compared a sample of N=858 members who enrolled during or after January 2021 in the digital DPP (date the digital DPP started enrolling individuals on Medicaid) to the broader US population with Medicaid coverage. After filtering out members who belonged to 12 census blocks with ADI suppression codes (1.7% of the available block groups in this sample), this resulted in a final population of N=844 digital DPP members with Medicaid insurance.

The Medicaid-insured comparator group contained N=28,559,532 US adults aged ≥19 years with only Medicaid/means-tested public coverage, or two or more types of health insurance coverage, including Medicare and Medicaid/means-tested public coverage. We estimated the total number of US adults on Medicaid to be 28,559,532, or 11.7% of the US adult population. We confirmed the accuracy of this estimate by calculating the percentage of adults and children on Medicaid as either their sole source of coverage or as part of their coverage to be 17.0% of the US population, very close to the 17.8% published by the Census Bureau for 2020 (28). After filtering out members who belonged to 2,878 census blocks with ADI suppression codes (1.3% of the available block groups in this sample), this resulted in a final population of N=28,247,337 adults with Medicaid insurance, or 11.6% of the US adult population.

### 2.5 Generating the ADI distributions for the digital DPP and US comparison groups

#### 2.5.1 Digital DPP members with commercial insurance

To generate the ADI distribution for the digital DPP members with commercial insurance, we connected each member’s census block group to the national percentile of block group ADI values by matching on the block group census ID (i.e., 12-digit FIPS code). After removing the census blocks with ADI suppression codes as indicated above, the national percentile distribution of block group ADI values for digital DPP members with commercial insurance represented 31,659 census block groups across 50 states and Washington, D.C.

#### 2.5.2 US population with commercial insurance

To generate the ADI distribution for the US population with commercial insurance, we connected the national percentile of block group ADI values to each US census block group in Table B27010 by matching on the block group Census ID (i.e., 12-digit FIPS code). After removing the census blocks with ADI suppression codes, the national percentile distribution of block group ADI values for US adults with commercial insurance represented 233,858 census block groups across 50 states and Washington, D.C.

#### 2.5.3 Digital DPP members with Medicaid coverage

To generate the ADI distribution for the digital DPP members with Medicaid, we connected each member’s census block group to the national percentile of block group ADI values by matching on the block group census ID (i.e., 12-digit FIPS code). After removing the census blocks with ADI suppression codes, the national percentile distribution of block group ADI values for DPP members on Medicaid represented 689 census block groups across seven states and Washington, D.C.

#### 2.5.4 US population with Medicaid coverage

To generate the ADI distribution for the US population with Medicaid, we connected the national percentile of block group ADI values to each U.S. census block group in Table B27010 by matching on the block group Census ID (i.e., 12-digit FIPS code). After removing the census blocks with ADI suppression codes, the national percentile distribution of block group ADI values for US adults on Medicaid represented 214,659 census block groups across 50 states and Washington, D.C.

### 2.6 Body mass index

For the digital DPP samples, each member’s starting weight was their first confirmed digital weight provided via the Lark-provisioned, connected scale within the first 30 days of their program. Members excluded from the body weight analyses did not manually confirm their weight or provided only a manual weight (i.e., not measured via the Lark scale). There were 29,162 digital DPP members with commercial insurance and 523 members with Medicaid who provided a first digital weight for the analyses assessing the relationship between ADI and starting body mass index (BMI; kg/m^2^). We categorized BMI based on CDC’s classification system (29): normal weight (<25 kg/m^2^), overweight (≥25 to <30 kg/m^2^), obesity class 1 (≥30 to <35 kg/m^2^), obesity class II (≥35 to <40 kg/m^2^), and obesity class III (≥40 kg/m^2^).

### 2.7 Statistical analyses

We used Python version 3.8.10 to conduct all statistical analyses. Histograms and cumulative histograms show the ADI distributions with 5% ADI value bin widths for the commercially insured and Medicaid populations, respectively. Two-sample Kolmogorov-Smirnov (KS) tests compared the two commercially insured sample distributions and two Medicaid sample distributions, respectively, for statistical differences. One-way ANOVAs tested whether the mean BMI values for each ADI quintile were equal for the commercially insured and Medicaid-covered digital DPP samples, respectively. An *a priori p* value <.05 denoted statistical significance for all analyses.

## 3 Results

### 3.1 Participant demographics and characteristics

The mean age of the digital DPP members with commercial insurance was 47.5 years (*SD* 10.6). The sample was 78.5% female, with an average starting weight of 101.3 kg (*SD* 23.9), height of 167.1 cm (*SD* 9.8), and BMI of 36.3 kg/m^2^ (*SD* 7.8) falling into class II obesity. Most members, 60.5%, identified as White/Caucasian, with 10.7% identifying as Black/African American, 3.3% as Asian, 0.5% as Native American, 0.3% as Pacific Islander, 9.9% selecting Other, and 14.9% choosing not to disclose this information. Regarding ethnicity, 77.6% of the sample identified as not Hispanic or Latino, 8.7% as Hispanic or Latino, and 14.9% chose not to report this information.

The mean age of the digital DPP members with Medicaid coverage was 42.2 years (*SD* 11.2). The sample was 80.1% female, with an average starting weight of 104.2 kg (*SD* 25.9), height of 166.8 cm (*SD* 9.9), and BMI of 37.3 kg/m^2^ (*SD* 8.6) falling into class II obesity. Most members, 73.2%, identified as White/Caucasian, with 9.3% identifying as Black/African American, 0.6% as Asian, 1.0% as Native American, 3.3% selecting Other, and 12.2% choosing not to disclose this information. Regarding ethnicity, 86.4% of the sample identified as not Hispanic or Latino, 1.4% as Hispanic or Latino, and 12.2% chose not to report this information.

Although Table B27010 in the 2016–2020 ACS for the US population samples did not provide full demographic information, we can report the breakdown of the overall population by age category. Adults aged 19–34 years represented 21.6% of the population included in these estimates, adults aged 35–64 years represented 38.3%, and adults aged 65 and older represented 15.9%.

### 3.2 ADI distribution comparisons

**Figure 1:**
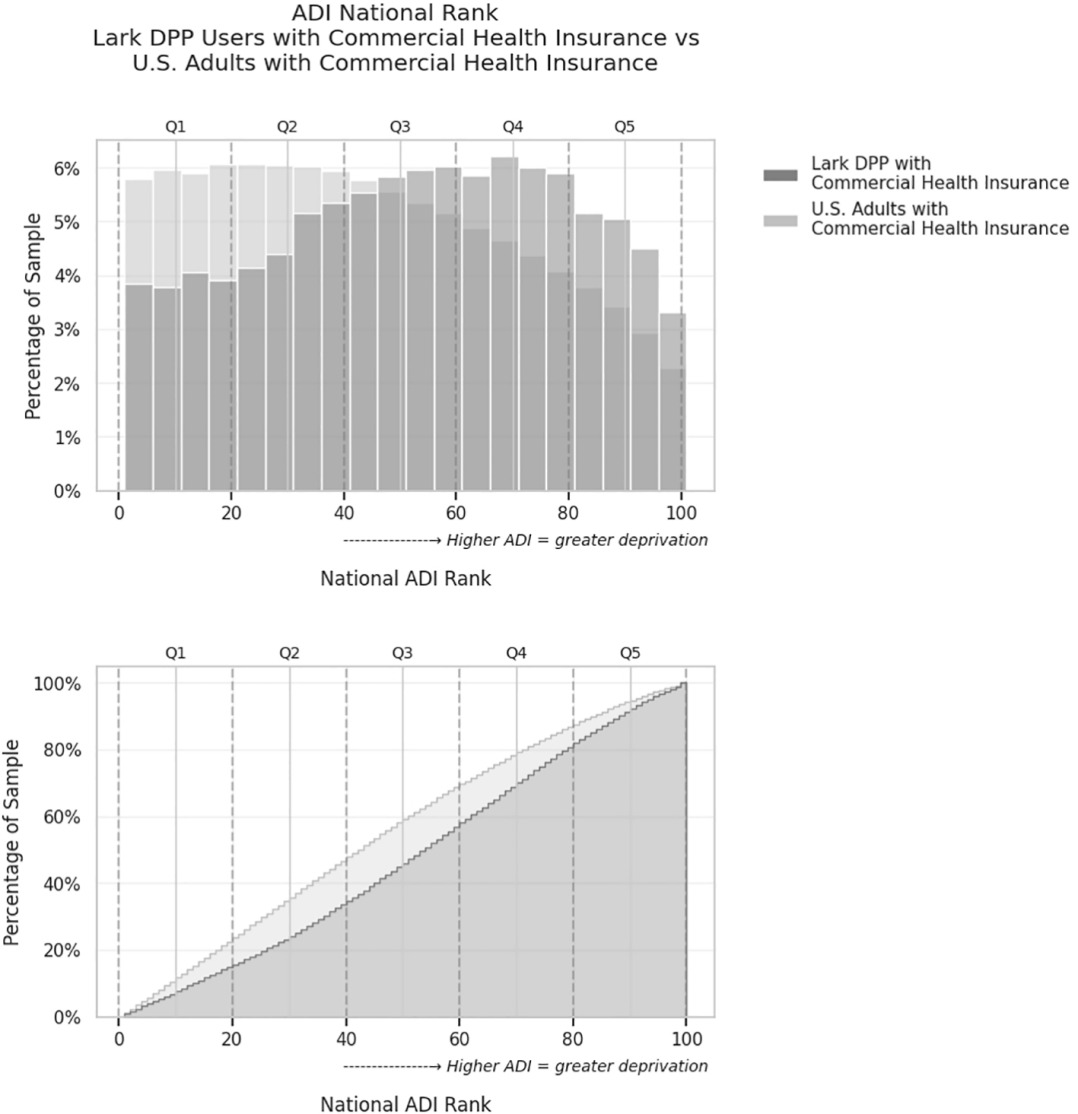
Comparison of Area Deprivation Index (ADI) national rank distributions for commercially insured Lark DPP users versus the overall U.S. commercially insured adult population. Upper: Histogram showing the percentage of individuals in each 5-point ADI bin. Lower: Cumulative distribution plot showing the proportion of individuals living in areas with ADI less than or equal to each value. Each point on the curve represents the percentage of the sample that lives in areas with an ADI rank less than or equal to a given value. The Lark DPP group has a greater proportion of users residing in higher-ADI (more deprived) areas, as indicated by the upward shift in the cumulative curve. Dashed lines denote quintile cutoffs for ADI (Q1 = least deprived, Q5 = most deprived).

**Figure 1** displays the distribution of these two samples across different ADI levels, with greater proportions of digital DPP living in higher ADI (more deprived) areas compared to the commercially insured comparison population. KS testing on the two distributions indicated that members of the digital DPP with commercial insurance lived in areas with a significantly higher ADI than the comparable commercially insured US population (KS test *D*=0.13, *p*<.001). Breaking the distribution into quintiles, the population of digital DPP members living in an area with an ADI in quintile 1 (least deprived) was 14.7% (6,100/41,375) vs. 22.5% (42,406,938/188,609,136) for the US population; quintile 2 was 18.7% (7,739/41,375) digital DPP vs. 24.1% (45,486,327/188,609,136) US; quintile 3 was 23.3% (9,645/41,375) digital DPP vs. 22.0% (41,427,483/188,609,136) US; quintile 4 was 24.0% (9,930/41,375) digital DPP vs. 18.2% (34,367,405/188,609,136) US; and quintile 5 (most deprived) was 19.2% (7,961/41,375) digital DPP vs. 13.2% 13.2% (24,920,983/188,609,136) US.

**Figure 2:**
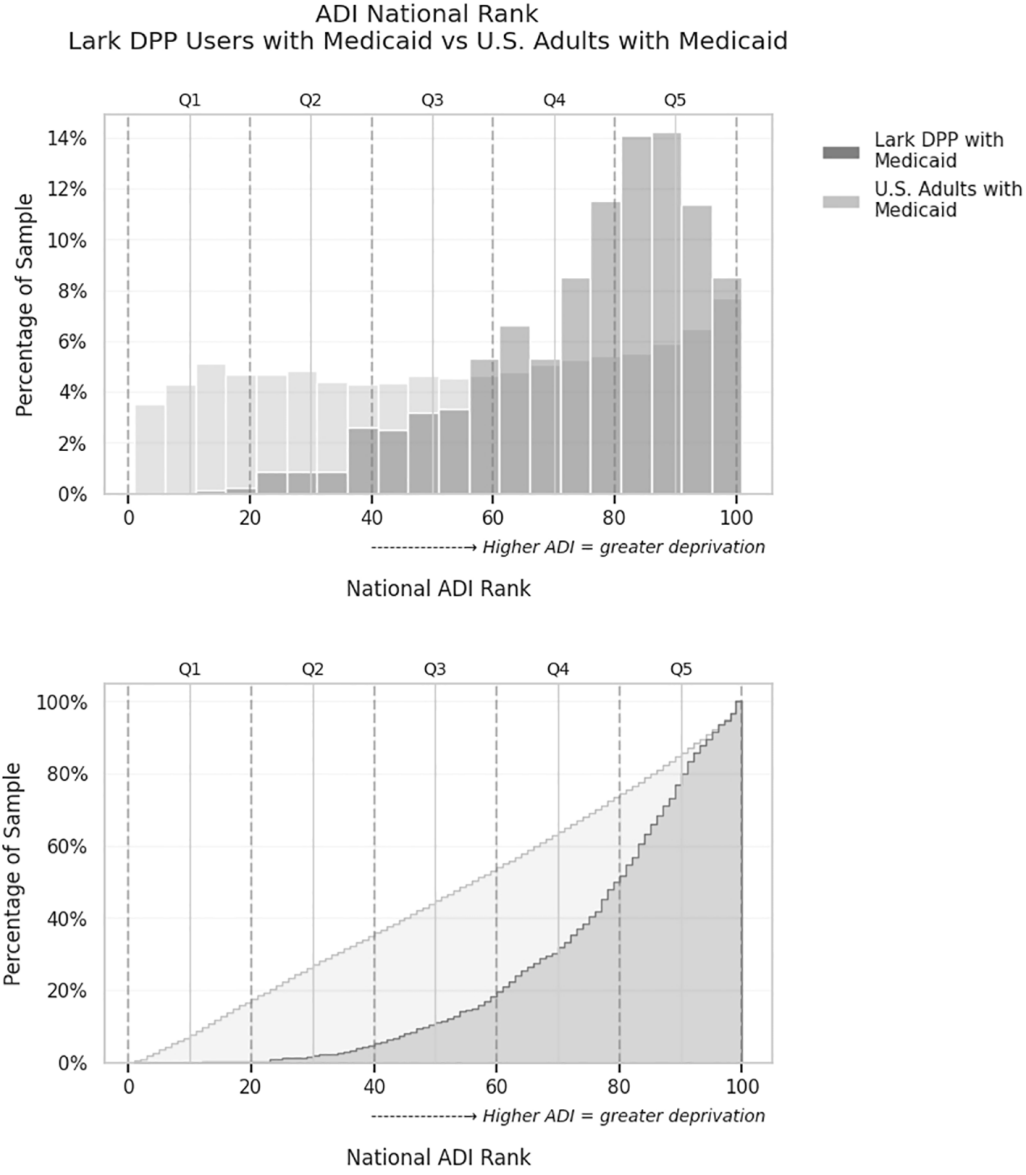
Comparison of Area Deprivation Index (ADI) national rank distributions for both the Medicaid-covered Lark DPP users versus the overall U.S. commercially insured adult population. Upper: Histogram showing the percentage of individuals in each 5-point ADI bin. Lower: Cumulative distribution plot showing the proportion of individuals living in areas with ADI less than or equal to each value. Each point on the curve represents the percentage of the sample that lives in areas with an ADI rank less than or equal to a given value. The Lark DPP group has a greater proportion of users residing in higher-ADI (more deprived) areas, as indicated by the upward shift in the cumulative curve. Dashed lines denote quintile cutoffs for ADI (Q1 = least deprived, Q5 = most deprived).

**Figure 2** displays the distribution of each of these samples across different ADI levels, with greater proportions of digital DPP living in higher ADI (more deprived) areas compared to the Medicaid-insured comparison population. KS testing on the two distributions indicated that members of the digital DPP with Medicaid coverage lived in areas with a significantly higher ADI than the comparable Medicaid-insured US population (KS test *D*=0.35, *p*<.001). Breaking the distribution into quintiles, the population of digital DPP members on Medicaid living in an area with an ADI in quintile 1 (least deprived) was 0.4% (3/844) vs. 16.7% 16.7% (4,704,890/28,247,377) for the US Medicaid population; quintile 2 was 4.4% (37/844) digital DPP vs. 18.3% (5,180,772/28,247,377) US; quintile 3 was 13.6% (115/844) digital DPP vs. 18.0% (5,092,094/28,247,377) US; quintile 4 was 31.6% (267/844) digital DPP vs. 20.3% (5,747,492/28,247,377) US; and quintile 5 (most deprived) was 50.0% (422/844) digital DPP vs. 26.6% (7,522,129/28,247,377) US.

### 3.3 ADI vs. BMI for digital DPP members

**Figure 3:**
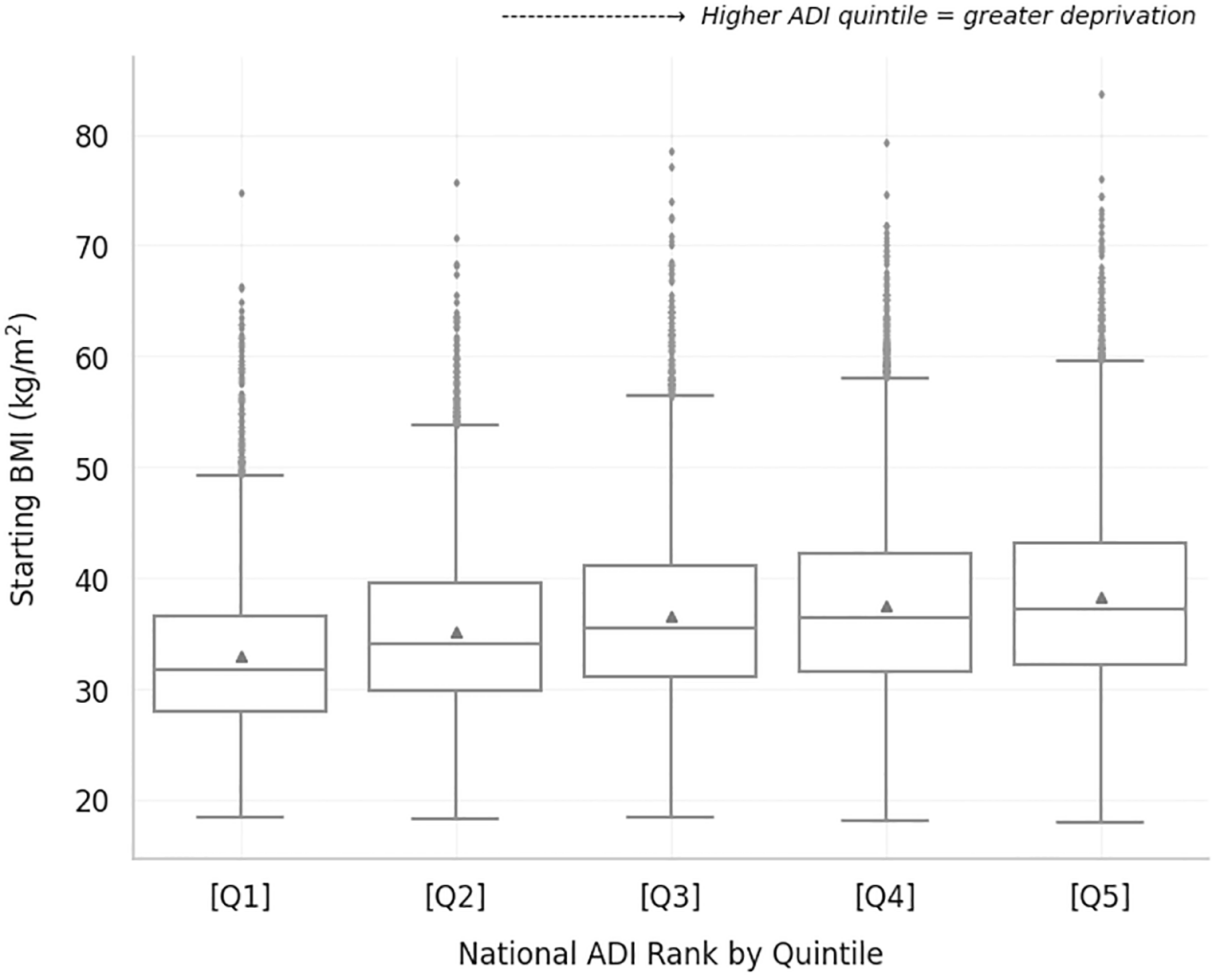
Starting BMI of Lark digital DPP members with commercial insurance for each ADI quintile. Average BMI is indicated by the triangle next to the median line for each boxplot.

As expected, given the CDC inclusion criteria for the DPP, almost all digital DPP commercially insured members with available weights were either overweight or obese. Only 3.1% (897/29,162) of the population had a healthy weight based on their first digitally provided weight. Overweight members comprised 19.0% (5,549/29,162) of the sample, obesity class I comprised 27.8% (8,111/29,162), obesity class II 21.7% (6,329/29,162), and obesity class III 28.4% (8,276/29,162). The average BMI was statistically different across ADI quintiles, *F(4, 32,006)*=395.8, *p*<.001. **Figure 3** shows that average BMI increases as ADI quintile increases (**Figure 3**), where the average BMI of members in quintile 1 (least deprived) was 32.9 kg/m^2^ (*SE*.10) vs. 38.2 kg/m^2^ (*SE*.11) in quintile 5 (most deprived). All Tukey-corrected *post hoc* comparisons of BMI between ADI quintiles were statistically significant (*p*<.05).

For the digital DPP Medicaid-insured members, 4.2% (22/523) were classified as healthy weight, 14.7% (77/523) were overweight, 24.7% (129/523) were obese class I, 21.4% (112/523) were obese class II, and 35.0% (183/523) were obese class III. In contrast to the commercially insured members, the average BMI was not statistically different across ADI quintiles, *F(4, 549)*=1.4, *p*=.2.

## 4 Discussion

This study compared the ADI of members participating in a fully digital delivery of the National DPP called the Lark digital DPP to that of US adults with either commercial insurance coverage or Medicaid coverage, respectively. In support of the primary hypothesis, members of the digital DPP with commercial insurance had a higher ADI relative to US adults with commercial insurance. Contrary to expectations, the digital DPP members with Medicaid coverage also had a higher ADI relative to US adults with Medicaid coverage. Finally, consistent with expectations, there was a higher average BMI with increasing ADI quintile for digital DPP members with commercial insurance. However, there was no increasing relationship between BMI and ADI for the digital DPP members with Medicaid coverage.

Population health outcomes and value-based care are important healthcare targets that require attention to SDOH. Digital health solutions can support essential functions of health systems and facilitate improved outcomes and value-based care (30). Consistent health system interactions may reduce the impact of SDOH on outcomes such as poor glycemic control and emergency department visits (31). Digital health solutions offer a way to support continuous healthcare interactions and exist along the full spectrum of the health continuum; however, there have been more disease management solutions with fewer resources dedicated to prevention efforts (32). A fully digital version of the National DPP offers access to this well-validated prevention program to individuals who may otherwise face barriers to participation. Indeed, digital program offerings have greatly increased enrollment in the National DPP in recent years (18). The results of this study demonstrate that in addition to increasing enrollment in the National DPP, a fully digital program can target and recruit individuals at high risk for type 2 diabetes living in deprived areas.

It has historically been challenging to reach and enroll individuals living in deprived areas into the National DPP, and programs that have high uptake can have a big impact on lowering diabetes risk in a population (33). Digital DPP members with commercial insurance living in the most deprived ADI quintiles 4 and 5 comprised 43.2% of the population in this study, compared to only 31.4% of US adults. Strikingly, 81.6% of digital DPP members with Medicaid coverage lived in ADI quintiles 4 and 5 compared to only 47.0% of US adults on Medicaid. Members living in these high-risk areas also exhibited increased risk factors for diabetes based on high BMI. Although we only observed an increasing relationship between BMI and ADI for members with commercial insurance, this is likely because the average BMI of the members with Medicaid coverage was higher overall, with 35.2% in obesity class III at program start.

It was somewhat surprising that such a high proportion of digital DPP members on Medicaid coverage residing in ADI quintiles 4 and 5 initiated the program. Previous reports in the literature have linked poor socioeconomic status, health literacy, and higher ADI to a higher no-show likelihood for a different version of digital healthcare – telehealth visits (34, 35). Potential reasons for no-shows could be a lack of internet access or familiarity with technology. However, prior research has also shown that when given the opportunity to engage with digital technologies like telehealth, even Medicare beneficiaries residing in disadvantaged neighborhoods increased telemedicine use during the COVID-19 pandemic (36). In fact, the highest odds of utilization were in people residing in the most disadvantaged neighborhoods, demonstrating that telehealth met an important need for these individuals. The present findings similarly support the use of digital health solutions for individuals living in disadvantaged neighborhoods by demonstrating that these solutions can reach individuals in deprived areas, despite potential challenges. Individuals living in deprived areas had the necessary technology (a smartphone) to download the application and the ability to follow directions and initiate use. This is a good indication that digital health interventions can serve the needs of deprived communities if appropriately tailored for these populations.

A major risk factor for diabetes is poor dietary habits (37). Education, resources, and support related to improving diet quality may be one important way to tailor an intervention for populations residing in deprived areas. Thomas and colleagues (38) showed that young people residing in more deprived areas of the UK were more likely to consume high-fat and sugar foods, had increased exposure to advertising for unhealthy foods, and had poorer awareness of health conditions associated with being overweight or obese. Although the National DPP provides nutrition education as a standard part of the PreventT2 curriculum, members may need continuous support between lessons to embrace these difficult behavior changes. Fully digital programs powered by AI offer members on-demand coaching that is immediate and relevant to their behaviors. For example, the immediate coaching provided by Lark focuses on the nutritional composition of meals rather than just calories (e.g., your meal had good amounts of whole grains, protein, and fruit but also had significant added sugars). Members receive feedback on how they can round out their day by incorporating additional healthy food groups (e.g., try to get more veggies and healthy fats as the day progresses). Immediate feedback is highly relevant to unique member actions and can support members in making better dietary choices each day. Continuous, tailored support of this nature is a unique advantage of a digital health solution that can meet members when and where they require assistance, rather than the less frequent or asynchronous feedback offered by other programs.

### 4.1 Strengths and limitations

This study leveraged large datasets for both the digital DPP and the comparator US population. The fully digital DPP had member representation in all 10 HHS regions, enabling comparison to the entire US population. This study assumes independence of observations for statistical tests, including the KS test. However, some geographic clustering may be present among participants residing in the same census block groups, which could impact the independence assumption. Although the large sample and broad geographic representation may mitigate this concern, we acknowledge it as a limitation.

Additionally, the distribution testing did not adjust for confounders, such as age or gender, because the KS test does not allow for adjustment for confounders and the comparison data from the ACS did not provide full demographic information that could be used for sensitivity analyses. We did not elect to examine complex interactions between the ADI and variables like race/ethnicity due to the relatively low representation of minorities among the DPP members. Notably, the digital DPP did not require members to report on their race and ethnicity, meaning that these data were not complete for these variables. However, interactions between race and ADI have been previously demonstrated; for example, racial minorities living in areas of high ADI have even worse reported cardiometabolic outcomes (39). A critical area of future research will be to better understand the relatively low uptake of digital health solutions among people belonging to racial and ethnic minorities. It is also crucial for digital DPPs to bolster efforts to increase racial and ethnic diversity in digital DPPs. For example, one new measure taken by the Lark digital DPP after this study was completed was providing Spanish language programming to make the program accessible to those who speak Spanish as their first language. Another strategy is to work with the digital DPP’s health partners (e.g., health insurance companies) on priority outreach to underserved groups within that health partner’s population.

Because participation in digital health programs is voluntary, our findings may be influenced by self-selection bias—individuals who choose to engage may differ systematically from those who do not. Additionally, this observational study cannot establish causal relationships between ADI and program participation but rather describes patterns of access and reach across socioeconomic strata.

Finally, this study did not assess the relationship between ADI and program outcomes. The effectiveness of digital health interventions may vary across different groups. Our primary interest for this study was the uptake of a fully digital DPP among individuals living in areas with high deprivation, and we intend to investigate how the ADI impacts outcomes achieved in future work.

### 4.2 Conclusions

Members of a fully digital DPP designed for individuals at high risk of type 2 diabetes lived in areas with higher ADI values compared to the US population. This finding was true for both members with commercial health insurance and for members with Medicaid coverage. Thus, a fully digital DPP demonstrated uptake in individuals residing in deprived areas with high rates of obesity, poverty, unemployment, low education, and limited access to health care. Providing much-needed, on-demand preventive care to individuals in these areas may help to mitigate the negative health impacts of living in a deprived area.

## Data Availability

The dataset analyzed during this study is not publicly available because it includes sensitive, potentially identifiable information that was obtained through a collaboration between Lark Health and its healthcare partners; data may be available from the corresponding author on reasonable request.
